# Trials and tribulations of recruiting 2,000 older women onto a clinical trial investigating falls and fractures: Vital D study

**DOI:** 10.1186/1471-2288-9-78

**Published:** 2009-11-25

**Authors:** Kerrie M Sanders, Amanda L Stuart, Elizabeth N Merriman, Meaghan L Read, Mark A Kotowicz, Doris Young, Roderick Taylor, Ian Blair-Holt, Alistair G Mander, Geoffrey C Nicholson

**Affiliations:** 1 Department of Clinical and Biomedical Sciences; Barwon Health, The University of Melbourne, Geelong, Australia; 2 Department of General Practice: The University of Melbourne, Melbourne, Australia; 3 General Practitioner, Geelong, Australia; 4 Geriatrician, Geelong, Australia

## Abstract

**Background:**

Randomised, placebo-controlled trials are needed to provide evidence demonstrating safe, effective interventions that reduce falls and fractures in the elderly. The quality of a clinical trial is dependent on successful recruitment of the target participant group. This paper documents the successes and failures of recruiting over 2,000 women aged at least 70 years and at higher risk of falls or fractures onto a placebo-controlled trial of six years duration. The characteristics of study participants at baseline are also described for this study.

**Methods:**

The Vital D Study recruited older women identified at high risk of fracture through the use of an eligibility algorithm, adapted from identified risk factors for hip fracture. Participants were randomised to orally receive either 500,000 IU vitamin D_3 _(cholecalciferol) or placebo every autumn for five consecutive years. A variety of recruitment strategies were employed to attract potential participants.

**Results:**

Of the 2,317 participants randomised onto the study, 74% (n = 1716/2317) were consented onto the study in the last five months of recruiting. This was largely due to the success of a targeted mail-out. Prior to this only 541 women were consented in the 18 months of recruiting. A total of 70% of all participants were recruited as a result of targeted mail-out. The response rate from the letters increased from 2 to 7% following revision of the material by a public relations company. Participant demographic or risk factor profile did not differ between those recruited by targeted mail-outs compared with other methods.

**Conclusion:**

The most successful recruitment strategy was the targeted mail-out and the response rate was no higher in the local region where the study had extensive exposure through other recruiting strategies. The strategies that were labour-intensive and did not result in successful recruitment include the activities directed towards the GP medical centres. Comprehensive recruitment programs employ overlapping strategies simultaneously with ongoing assessment of recruitment rates. In our experience, and others direct mail-outs work best although rights to privacy must be respected.

**Trial registration:**

ISRCTN83409867 and ACTR12605000658617.

## Background

It is widely acknowledged that falls and fractures among older population are a major health problem and are associated with significant morbidity and reduced quality of life in Australia [1-4] and in other developed populations [5,6]. The highest rate of fracture and the highest number of fractures occur in the oldest sector (70 years and over) of the female population where 3-5% of the older population sustain a fracture each year [7,8]. Almost 90% of all hip fractures result from the impact of a fall [9]. Randomised, placebo-controlled trials are needed to provide evidence demonstrating safe, effective interventions that reduce falls and fractures in the elderly.

Intervention studies with a fracture outcome require large sample sizes of older adults yet frail older women are difficult to recruit onto placebo-controlled trials of long duration. Power calculations suggest 6,500 to 7,000 person-years of intervention are needed to detect a 22% difference in fracture rates between the active and placebo arms of the study population of women aged 70 years and older [10]. The quality of a clinical trial is dependent on successful recruitment of the target participant group. Valuable time and financial resources can be wasted when recruitment efforts are ineffective. This paper documents the successes and failures of recruiting over 2,000 women aged at least 70 years and at higher risk of falls or fractures onto a placebo-controlled trial of six years duration. The characteristics of study participants at baseline are also described for this study that investigated the role of an annual large dose of oral vitamin D_3 _in reducing falls and fractures in older women. The single-centre study was an investigator-initiated clinical intervention trial funded through competitive government research grants.

## Methods

### Aims

The Vital D Study aimed to demonstrate that an annual large-dose of vitamin D_3 _(500,000 IU) would prevent falls and fractures in older women who are at high risk of these events. Secondary aims of the study were to demonstrate that an annual large-dose of vitamin D (1) was a practical primary health care intervention (2) has a role in the prevention of depressive symptoms and (3) decreases total healthcare utilisation.

### Study Design

The Vital D Study was designed as a double blind, placebo-controlled trial in which 1,500 women at high risk of fracture are randomised to receive either 500,000 IU vitamin D_3 _(cholecalciferol) or placebo every autumn for five consecutive years.

The original protocol specified 1,500 women participating for 5.5 to 6 years allowing for up to 1750 person-years lost to withdrawal would achieve our target person-years of intervention. The protocol was later amended to expand recruitment to 2,300 participants. This new target would compensate for the 'slower than anticipated' rate of recruitment in the initial phases of the Vital D Study and allow the study goals to be achieved within the original time frame of the grant.

The initial 1500 participants recruited onto the study were largely residents of the local Barwon Statistical Division. This electoral division has a total population of 225,000 and consists of a large regional city (Geelong) surrounded by smaller coastal towns that have a significant proportion of older, retired residents. Recruitment of the extra 800 participants was largely targeted to the retired population of the Mornington Peninsula. This region is in the same state (Victoria) and similar latitude (38° South) but is located on the opposite side of the bay.

A nested group consisting of 137 participants consented to be studied more intensively over the duration of the Vital D Study. The design of this nested study will be described in detail in a subsequent paper.

The study was approved by the Barwon Health Human Research Ethics Committee (BH 02/60) and The University of Melbourne's Human Ethics committee (HREC No. 0200700). The study is registered with both the Australian Clinical Trial Registry (ACTR No. 12605000658617) and the International Standard Randomised Controlled Trial Number Register (ISRCTN No. 83409867). This research was carried out in compliance with the Helsinki Declaration.

### Study population

Women aged 70 years and older who fulfilled the defined criteria for increased risk of fracture were eligible for the study if they were not taking medication that affected bone and calcium metabolism at baseline, did not have renal disease, hypercalcemia, sarcoidosis, tuberculosis or lymphoma. Potential participants were excluded if their serum corrected calcium was greater than 2.65 mmol/L, serum creatinine greater than 150 umol/L or if their current medications included any of the following:- vitamin D greater than 400 IU/day; bisphosphonates, SERMs, HRT or calcitriol. The target group was community dwelling women so volunteers were excluded if their current permanent residence at recruitment was a high-level care facility. Participants were not withdrawn if they moved into high-level care during the course of the study.

Women were judged to be at an increased risk of fracture if they scored at least 5 points on an algorithm, adapted from identified risk factors for hip fracture (Table 1)[11].

**Table 1:** Eligibility algorithm for the Vital D Study: A total score of at least 5 points was needed to be eligible for participation in the study.

RISK FACTOR	GUIDELINES	SCORE	PATIENT SCORE
"Faller"	Self-reported or assessed as high risk of falling by the ***P****rimar****y C****are**** P****hysician*	5	
Any Fracture	Since age of 50 years	5	
High risk of low vitamin "D"	Avoids sun, wears protective/cultural clothing regularly uses sunscreen and/or have dark skin	5	
Osteoporosis	Bone density T-score at spine or hip >-2.5	5	
Osteopenia	Bone density T-score at spine or hip -1.0 to -2.5	2	
Aged > 80 years		2	
Maternal hip fracture		1	
Thinness	BMI < 20 or current weight less than that at age 25	1	
Self rated health as fair or worse	(excellent/good/fair/poor/very poor)	1	
Poor vision	Self reported or assessed by primary care physician	1	
No walking exercise	"Do you walk outside your house or yard regularly?"	1	
On feet < 4 hours per day		1	
Inability to rise from a chair	*without using arms*	1	
Caffeine intake	> Two cups of coffee or 4 cups of tea per day	1	
Smoker	Current	1	
Long-acting benzodiazepines	Current use	1	
Anticonvulsant	Current use	1	
History of thyrotoxicosis		1	
Resting pulse > 80/min		1	
	**SCORE ≥ 5 = eligible for Vital D Study *(Check exclusions listed below)***	**TOTAL**	

*EXCLUSIONS*			
*Hypercalcemia*	*Baseline serum corrected calcium > 2.65 mmol/L*		
*Bone-trophic medication*	*Current use; HRT, bisphosphonate, SERM, calcitriol, Vitamin D supplement > 400 IU/day*		
*Renal disease*	*Creatinine > 150 μmol/L or no known renal disease*		
*Sarcoidosis, TB or lymphoma*			

### Recruitment

A variety of recruitment strategies were employed to attract potential participants. These strategies were developed through past experience of recruiting for smaller clinical trials; from collaborating with other professionals who work with the elderly and from team meetings and 'brain storming' during reviews of our recruitment efforts. The recruitment strategies can be grouped into six categories:

1. Doctor/Health Professional referral

2. Club presentation

3. Church/Club newsletter

4. 'Word of mouth'

5. Media (newspaper/radio)

6. Targeted mail outs

Statistics were collected on method of recruitment through standardised questioning at initial contact with the potential participant.

#### 1. Doctor/Health Professional referral

The original study protocol aimed to recruit a majority of participants through their local general practitioner (GP) as the study was designed to be easily translated to clinical practice. If the study findings were positive it was envisaged that elderly women might receive their annual dose of vitamin D supplementation when they visited their GP to receive their annual influenza vaccination. To meet this goal the research team initially directed substantial recruitment efforts towards engaging the GPs. To help involve GPs in the Geelong region (n = 200), the research team obtained the guidelines for accreditation of Continuing Medical Education (CME) Activities from the Royal Australian College of General Practitioners. A program was developed and subsequently accredited by the College so that GPs could earn their CME activity points by attending a symposium of 'An Update on Vitamin D and Osteoporosis'. This was also an opportunity to present the study goals and involve the GPs in recruitment. The symposium was conducted in April 2003 and was followed up by the research team visiting the medical centres with brochures and posters for the waiting rooms. These visits were pre-booked with the Practice managers and included a ten-minute summary presentation on a laptop computer. Support staff at the medical practices were also encouraged to attend. The research team provided a light lunch as the meetings were commonly scheduled during the doctors' brief lunch break. To minimise the time commitment of the doctor, the algorithms were electronically loaded onto the practice medical software package so that it would automatically appear when the doctor treated any female patient aged at least 70 years and not currently on medication that affect bone and calcium metabolism. For doctors who did not want the electronic reminder, the study's algorithm was commercially printed onto 'tear-off' A4 notepads. If an older woman consented to be contacted by the research team the doctor merely had to adhere a patient's identification label to the algorithm sheet. The research team collected these algorithm pages from the administration staff at the practice and would telephone the potential participant to check eligibility criteria.

#### 2. Club presentation

The research team approached retirement villages, as well as service clubs and sporting facilities that typically cater for an older community. The team gave 35 visual presentations about vitamin D and the research project. Prior to each presentation the event was advertised in the clubs' newsletter and posters displayed in the clubrooms.

#### 3. Church/Club newsletter

The study advertisement was placed in newsletters that had a target audience of older females. Where possible the advertisement appeared in two or three consecutive club newsletters. These newsletters were often prepared and distributed by volunteers and the cost to the research team for the study advertisement was minimal.

#### 4. 'Word of mouth'

Once the study had several hundred women participating, the team organised morning teas for participants and invited them to bring a friend. This activity had two goals (1) to give participants a sense of 'belonging and ownership of the project' and (2) to provide an opportunity for the friend to potentially enrol in the study.

During the first year of the project, the research team set up an information stall at the local three-day agricultural show. To create interest at this stand printing the study logo give-aways such as pencils and sun visors was investigated but not used due to the prohibitive expense. Similarly, printing the study logo on milk cartons was also investigated but rejected on the basis of cost.

#### 5. Media (newspaper/radio)

The Vital D Study featured in local newspapers 12 times including advertisements (paid) and general interest articles (no charge). Advertisements were placed in the same paper consecutively for 2 to 3 weeks. The team also employed a public relations/media company (DolphinBewley^® ^Public Relations; Ocean Grove, Victoria) to review the study handouts and engage media coverage for recruitment. The study had a total of three interviews on radio including one national radio program. A toll-free phone number was set up so that potential participants were not discouraged by the cost of telephone calls.

#### 6. Targeted mail outs

The research team gained approval from several government agencies to have the recruitment brochures distributed to women aged 70 years and older on their database. In each case the study was invoiced for the cost of the mail-out. The research team supplied the generic brochures/letters and the agencies/government body collated and posted the information so that confidentiality was maintained with their clients. The recipient was asked to complete the enclosed form and return it in the reply-paid envelope if they were interested in obtaining more information. These targeted mail outs were done using the databases from (1) the local government council's recipients of meals-on-wheels and home help (n = 2,000), (2) the public regional hospital (n = 3,600), (3) a large rural medical centre (n = 800) and (4) Victorian electoral roll (n = 23,000).

In Australia voting is compulsory for all residents. The Victorian Electoral Commission (VEC) database contains all adult residents of the region and can be sorted by date of birth and postal code. The mail-out from the VEC occurred as two distinct tasks. In the first instance the VEC was supplied with postcodes of the Barwon Statistical Division and 15,000 women in the target age group received letters. The second VEC mail-out was targeted to 8,000 residents of the Mornington Peninsula.

In conjunction with the VEC mail out to the Mornington Peninsula, the research team sent a letter explaining the research project to all general practitioners in the region. Individual letters were not sent to doctors in the Barwon region as the Vital D Study had been advertised amongst the general practitioners in several other ways (CME activity, presentations at the medical centres and local paper advertisements).

### Randomisation and blinding

An independent statistician carried out computer randomisation of participants using their unique study identification numbers and the statistical software Minitab™ (version 13). The randomisation lists of 'active' and 'placebo' status were then directly emailed to the hospital's clinical trials pharmacist who was responsible for all dispensing of study medication throughout the trial.

## Results

### Recruitment

The Vital D study recruited for two years to reach a revised target of 2,300 participants (June 2003 to 2005). Of the 2,317 participants, 74% (n = 1716/2317) were consented onto the study in the last five months (February to June 2005). This was largely due to the recruiting success of a targeted mail-out from the VEC. Prior to this only 541 women were consented in the 18 months of recruiting to the end of 2004.

A total of 70% of all participants were recruited as a result of targeted mail-out (Table 2). The response rate from the letters averaged 7.6% [VEC (n = 1299/15000, 8.7%); local public hospital (n = 210/3600, 5.8%); Barwon local government aged care recipients (n = 63/2000, 3.2%); a large rural medical centre (n = 57/800, 7.1%)].

**Table 2:** Source of recruitment

Code	Recruitment Strategy	Eligible-consented	Eligible-not participating	Ineligible	Responded 'NO'*	Total Screened
1	Targeted mail outs.	70.3% (1629)	(372)	(347)	(2486)	67.1% (4834)
2	Doctor/Health Professional referral	5.0% (116)	(57)	(28)		2.8% (201)
3	Club presentation	1.5% (34)	(5)	(2)		0.5% (41)
4	Church/club newsletter	1.5% (37)	(10)	(12)		0.8% (59)
5	Word of mouth	2.2% (51)	(10)	(10)		1.0% (71)
6	Media (newspaper/radio)	1.3% (30)	(6)	(14)		0.7% (50)
7	Other.	18.1% (420)	(362)	(1166)		27.0% (1948)
	% consented participants	100%				

	% total number screened	32% (2317/7204)	11% (822/7204)	21.9% (1579/7204)	34% (2486/7204)	100% (7204)

* did not complete algorithm

The successful response rate from the mail out of the study brochure increased from 2 to 7% following revision of the material by a public relations company. The changes included a photo of older people replacing our photo of tablets, removing the word 'research' from the front cover of the brochure and some simplification of the language used. The order that the information was presented was also changed. The PR company charged the study at a discounted 'not for profit' hourly rate and also arranged several media - radio and print exposures for recruitment of participants onto the study.

Only 5% (n = 116/2,317) of the participants were recruited through their local GP medical centre (Table 2). Club presentations averaged only one eligible participant recruited per event (Table 2). The 'bring a friend' morning tea resulted in 30 new participants from 200 participants who attended the event. Another 30 participants were recruited through the media coverage. In total, 6.5% were recruited collectively through club presentations, church newsletters, word of mouth and media. The source of recruitment for 18% of participants (n = 420) was not documented. This includes at least 155 participants who contacted the study team but did not remember how they first heard about the study.

### Characteristics of participants from different recruitment methods and residential regions

Participant demographic or risk factor profile did not differ between those recruited by targeted mail-outs compared with other methods ('faller' p = 0.242, 'fracture prior to 50 years' p = 0.227, 'high risk for low vitamin D' p = 0.101 or 'osteoporosis' p = 0.597). The median age did not differ between those recruited by mail-out versus other methods (median {range} age 76.1 {70.0-91.3} and 76.2 {70-93.9} years, respectively; p = 0.345). There were no differences in the proportion of women recruited by targeted mail-out compared with other recruitment strategies between those 'eligible and did participate', those 'eligible but did not participate' and those 'ineligible' for the study (83%, 81% and 84%, respectively; p = 0.403).

### Participant characteristics (Table 3)

**Table 3:** Participant Characteristics

Characteristic	
Age - median (range), years	76.0 (70.0 - 93.9)
	
Total person-years	6925.2
	
Region	
- Barwon	71.3% (n = 1609)
- Mornington Peninsula	23.0% (n = 520)
- Other	5.6% (n = 127)
	
*Eligibility criteria (5 points)*	
Faller (self-report or local Dr) Yes	38.9% (n = 878/2256)
Prior fracture since 50 years	18.8% (n = 424/2256)
High risk of low vitamin D	36.3% (n = 819/2256)
Osteoporosis diagnosis	1.0% (n = 23/2256)
Totalled 5 from 1 & 2 point criteria	5.0% (n = 112/2256)

Thirty-nine percent of all participants were classified as 'faller' at recruitment (n = 878/2256) and 19% (n = 424/2256) earned their 5-point eligibility under the criteria of previous fracture since age 50 years. Another 36% (n = 819/2256) were classified as being at high risk of low vitamin D at baseline. Only 1% (n = 23/2256) of participants earned their 5-point eligibility through a self-report of osteoporosis. And only 5% (n = 112/2256) of participants gained eligibility to the study through multiple two and one point criteria that summed to five points.

At baseline the median age was 76.0 years (range: 70.0-93.9 years). The total intervention period for the study was 6,930 person-years with only 384 person-years lost to follow-up, which represents only 5.3% of the potential person-years (7314 p-y).

### Withdrawn Participants (Table 4)

**Table 4:** Withdrawn participants

	Participants withdrawn prior to any study medication	Participants withdrawn during intervention period
**Person-years lost to withdrawal**		**384.2**

	(n = 59)	(n = 226)
Death	8.5% (n = 5)	38.5% (n = 87)
Illness	17.0% (n = 10)	23.0% (n = 52)
Disinterest/too busy	10.2% (n = 6)	13.7% (n = 31)
Old age/dementia	11.9% (n = 7)	8.0% (n = 18)
Personal reasons/no reason	15.3% (n = 9)	6.2% (n = 14)
Discouraged by family or Doctor	6.8% (n = 4)	1.3% (n = 3)
Avoiding unnecessary tablets/tests	11.9% (n = 7)	2.2% (n = 5)
Met exclusionary criteria	11.9% (n = 7)	
Other	6.8% (n = 4)	7.0% (n = 16)

Number of participants in parenthesis

**Figure 1:**
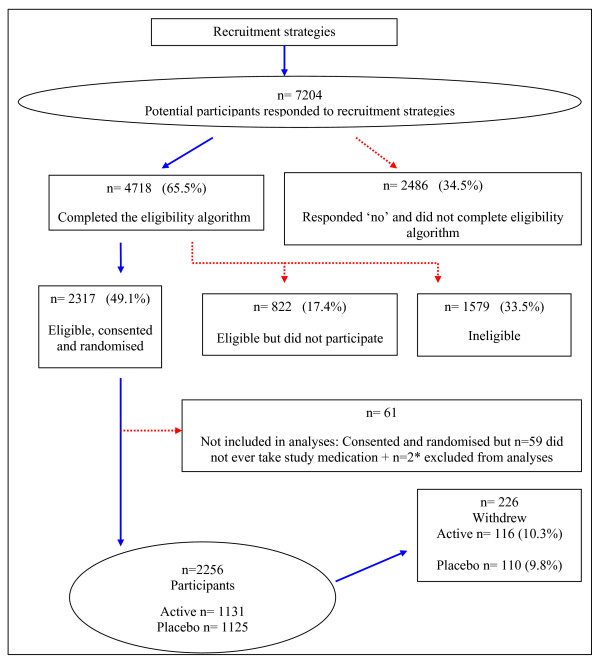
**Recruitment flow chart**. * Two participants were excluded from analysis due to a combination of failing memory and excessive number of falls, i.e. more than two per day.

As the annual dose of study medication was only administered during autumn and winter, some women were consented onto the study up to six months prior to receiving any study medication. A total of 59 women withdrew from the study during this period prior to their first dose of study medication. Their reasons for withdrawal included illness (17.0%), personal reasons/no reason (15.3%), old age/dementia (11.9%), wanting to avoid unnecessary tablets/tests (11.9%) and discouraged by family or doctor (11.9%). Two participants were also excluded from the analysis due to a combination of a failing memory and an excessive number of falls, i.e. more than two per day. Thus the number of participants was 2,256 (Figure 1).

Two hundred and twenty-six women withdrew from the study after receiving at least one dose of study medication. This represents 10% of Vital D participants. The most common reasons for early withdrawal from the study was death (38.5%), illness (23.0%), disinterest (13.7%) and inability due to old age or dementia (8.0%). A lower percentage of those recruited through targeted mail out later withdrew from the study (10.6% {n = 173/1629} vs. 16.3% {n = 112/688, targeted mail out vs. other recruitment methods, respectively; p < 0.001).

## Discussion

The most successful recruitment strategy was the targeted mail-out with 70% of all participants recruited by this method. The mail out from the VEC was so successful in the local Barwon/Geelong region that the team decided to expand the mail out to the Mornington Peninsula and increase the target number of participants from 1,500 to 2,300. This allowed the study to be completed in the original time frame compensating for an initial slow rate of recruitment. The mail-outs from the government agencies (VEC, databases from the local public hospital and local government home-help agency) each resulted in a similar recruitment rate of five to seven percent (% of participants recruited out of the total number of letters sent). The targeted mail-out from a large rural medical centre had a comparable response rate (7.1%).

A majority of participants resided in two distinct regions (Barwon/Geelong 69.4% and Mornington Peninsula 22.4%). The response rate from the mail-out was no higher in the local region (Barwon/Geelong) where the study had extensive exposure through other recruiting strategies. This was compared to the targeted mail-out at the Mornington Peninsula where no presentations took place. However the letter describing the study sent to all general practitioners in the Mornington region may have improved their response rate. Since participants were not asked if they had discussed the study with their GP we are unable to determine the influence of their local doctor on recruitment rates. General practitioners in the Barwon/Geelong region were informed of the study through advertisements in the regional GP Association's newsletter and the education forum offered to them as part of their continuing medical education requirement.

Engaging the services of a small public relations company enhanced the success of the mail-outs. The company revised the study brochure/information letter. Although the changes appeared minor, the positive response rate to the letter increased from 2 to 7%.

The 'bring a friend' morning tea offered to participants in the early stages of recruitment was also a worthwhile activity. The morning tea resulted in 30 new participants from the 200 invited participants.

The strategies that were labour-intensive and did not result in successful recruitment include the activities directed towards the GP medical centres. The team spent considerable time and effort (1) developing the accredited CME activity for GPs, (2) developing and installing the electronic version of the screening algorithm that was compatible with GP medical practice software and (3) giving approximately 25 presentations to medical staff. Although referral of potential participants from doctors and other health professionals comprised the second most successful recruitment strategy, the method was labour-intensive and only contributed 7.4% of all participants. The 'hands-on' approach of public and targeted presentations was similarly labour-intensive and while generating a lot of interest about osteoporosis and vitamin D, this approach averaged only one eligible participant per presentation.

A majority of the participants with a recruitment method classified as 'other or not recorded' are likely to have been contacted through the targeted mail-outs from VEC. This was a very busy period of recruitment and staff had little time to follow-up on the unanswered response relating to the recruitment method. Staffing levels during this recruitment period were 0.8 equivalent full time (EFT) research scientist, 0.3 EFT research nurse and 0.3 EFT administrative officer. At this time staff were also responsible for the monthly ascertainment of all falls and fractures of participants already enrolled onto the study.

Other recruitment reports of clinical trials have identified similar recruitment rates from direct mail-outs, 'word of mouth' and posters/brochures. Robinson and colleagues [12] enrolled 70 families into an obesity prevention trial for young children and report 66% were obtained through direct mail-outs; 7% from other sources such as 'word of mouth' and 3% from posters and brochures. However, in contrast to our findings, Robinson's team acquired 24% through newspaper advertisements compared with 1% of our participants. Earlier research suggests that newspaper advertisements and media can promote widespread awareness and a positive image of the study [13]. While casual feedback from participants suggests this is true, our similar recruitment rate for the direct mail-outs in the Mornington region would suggest our advertising strategy in the local region had little additional impact on the results from the direct mail-out. The discrepancy in advertising success between the Vital D study and Robinson's trial may be explained by the different age of participants. Older people are more likely to have a close relationship with their local doctor while their vision and age may impede their ability to be influenced by advertisements in the local newspaper.

Our rate of recruitment is consistent with that of an intervention trial on vitamin E in adults aged 50 to 80 years. The VECAT study recruited 1,204 participants in 60 weeks [14]. Similarly the Vital D team recruited 2,317 older women over 104 weeks, although 74% were recruited in the last 20 weeks. The lessons learnt from the initial poor response rate of Vital D recruitment include changing strategies earlier when the initial efforts are unsuccessful. As the study's secondary aim was to demonstrate that this was a practical primary health care intervention the research team were reluctant to minimise the GP's involvement in recruitment. Garrett's study on vitamin E also reported a lack of recruiting success with direct approaches to community groups and via general practitioners [14]. Although these researchers suggest their low response rate from GPs may be due to weak collaborative links and no follow-up with the doctors by study personnel, these reasons are not applicable in the Vital D study. The team had (1) strong links with a majority of the 200 GPs in the local region, (2) a Chief Investigator that was also Head of the department of General Practice at The University of Melbourne and (3) an Associate Investigator who was president of the local GP Association.

Garrett and colleagues also reported that recruiting via direct mail-out using the electoral roll was efficient and cost-effective. Their recruitment rate of 5% from this method was in agreement with our rate of 7%. Although Garrett's research team observed that newspaper advertising was equally cost-effective as the electoral roll mail-out, their high initial response could not be duplicated in subsequent advertisements [14].

The study has a high retention rate with few participants choosing to withdraw. The total person-years lost was only 5.3% of the maximum possible person-years (7,309 person-years) with 3.9% (n = 87/2256) of participants dying during the course of the study. Casual feedback from participants suggests that retention rates were improved by (1) regular phone contact by the same team members, (2) regular newsletters that included 'fun' staff profiles and (3) Christmas cards.

The study was designed to investigate the effect of an annual dose of vitamin D in older women at high risk of falls and fractures. The eligibility criteria of 5 points scored on the screening algorithm aimed to identify high-risk women. Successful recruiting involves both reaching the target number of participants and selecting individuals with the appropriate risk profile that satisfies the original power calculations. While it can be tempting to accept eager potential participants who are in the correct age range, the 'healthy volunteer' bias is well-documented [15]. Based on our eligibility algorithm 22% of potential participants screened were rejected thus hopefully eliminating the 'healthy volunteer' bias.

## Conclusion

It is common for researchers to face recruitment problems [16] and a successful clinical trial is dependent on recruitment. As reported by other studies, the Vital D team found it useful to conduct regular meetings to review recruitment strategies and generate new ideas. Comprehensive recruitment programs employ overlapping strategies simultaneously [17] with ongoing assessment of recruitment rates [12]. In our experience, and others [12,14] direct mail-outs work best although rights to privacy must be respected.

## Competing interests

The authors declare that they have no competing interests.

## Authors' contributions

KMS was involved in all stages of the study including study design, analysis and writing the manuscript. ALS participated in study coordination and analysis. ENM was involved in study coordination and analysis. MLR was the study coordinator during most of the recruitment period. MAK was involved in study design. DY was involved in instigating recruitment strategies targeted to GPs. RT, IB-H and AGM are medical practitioners who were actively involved in incorporating recruitment strategies targeted to GPs. GCN has played a leadership role in all stages of the study from design to manuscript submission.

All authors participated in manuscript preparation including reviewing and approving the final manuscript.

## Pre-publication history

The pre-publication history for this paper can be accessed here:

http://www.biomedcentral.com/1471-2288/9/78/prepub
